# A Degradable Nanosystem Based on Small Gold Nanoparticles and Albumin for Amyloid Aggregation Inhibition

**DOI:** 10.3390/pharmaceutics18040504

**Published:** 2026-04-19

**Authors:** Matías Levio, Francisco Rossel Carrera, Fredys Sánchez Hoyos, Maycol Huerta, Carlos Alamos, Rodrigo Vásquez-Contreras, Marcelo J. Kogan, Eyleen Araya Fuentes

**Affiliations:** 1 Facultad de Ciencias Quimicas y Farmacéuticas, Universidad de Chile, Sergio Livingstone 1007, Santiago 8380492, Chile; matias.levio@ug.uchile.cl (M.L.); frosselcarrera@gmail.com (F.R.C.); fsanchezh@unicartagena.edu.co (F.S.H.); labkogan@gmail.com (C.A.); 2 Departamento de Ciencias Quimicas, Facultad de Ciencias Exactas, Universidad Andres Bello, Republica 275, Santiago 8370146, Chile; m.huertamatus@uandresbello.edu (M.H.); rvasquez1982@gmail.com (R.V.-C.); 3 Advanced Center for Chronic Diseases (ACCDiS), Facultad de Ciencias Quimicas y Farmaceuticas, Universidad de Chile, Dr. Carlos Lorca Tobar 964, Santiago 8380494, Chile

**Keywords:** degradable nanoparticles, near-infrared irradiation, beta amyloid

## Abstract

**Background/Objectives**: Beta amyloid (Aβ) aggregates play a central role in the pathophysiology of Alzheimer’s disease (AD), and their detection and modulation remain major challenges in developing effective therapeutic and diagnostic strategies. Previously, gold nanoparticles with plasmonic and optical properties in the near-infrared (NIR) region and photothermal capabilities have been designed for detecting and disaggregating Aβ aggregates. However, these systems often face limitations related to biodegradability, long-term accumulation, and safety. In this work, a degradable NIR-responsive nanosystem based on small gold nanoparticles (sAuNPs), potentially excretable due to their small size, encapsulated within bovine serum albumin (BSA) and functionalized with the all-D peptide D3, was developed to inhibit Aβ aggregation. **Methods**: sAuNPs (~5–6 nm), functionalized with HS-PEG-NH_2_, were encapsulated into BSA nanoparticles using a desolvation method and subsequently conjugated to D3, resulting in the nanosystem f-sAuNPs-BSANPs-D3. The nanosystem was characterized by UV–Vis–NIR spectroscopy, dynamic light scattering, zeta potential analysis, electron microscopy, and nanoparticle tracking analysis. The effects of the nanosystem on Aβ_1–42_ aggregation were evaluated using a thioflavin T assay and electron microscopy. Additionally, the effects of f-sAuNPs-BSANPs-D3 on cell viability and its stability against trypsin digestion were assessed. **Results**: The nanosystem exhibited a measurable photothermal response under NIR irradiation and significantly reduced fibril formation. It did not affect the viability of SH-SY5Y neuronal cells at the tested concentrations. Trypsin incubation experiments demonstrated that the nanosystem remained stable at low enzyme concentrations mimicking plasma conditions, whereas higher enzyme concentrations induced degradation of the albumin matrix and subsequent disaggregation of sAuNPs. **Conclusions**: Overall, this study presents a degradable, albumin-based sAuNP nanosystem with NIR-responsive properties and potential for nanomedicine applications to inhibit Aβ aggregation in AD.

## 1. Introduction

Alzheimer’s disease (AD) is the most common form of dementia, accounting for nearly two-thirds of cases in individuals over 65. Pathologically, AD is characterized by toxic amyloid-β (Aβ) aggregates and neurofibrillary tangles (NFTs) made of tau protein [1]. Detecting and breaking down these aggregates are key in the theranostic (therapy plus diagnosis) approach to AD. Plasmonic nanoparticles have become promising tools for diagnosis and treatment because of their localized surface plasmon resonance (LSPR) and photothermal properties. LSPR enhances detection, while their ability to absorb light and generate heat helps disaggregate toxic Aβ aggregates [2]. These nanoparticles improve biomarker detection, aid in imaging and breaking down Aβ aggregates, and can deliver drugs to the brain. They also protect therapeutic molecules from degradation, making them a valuable platform for combined diagnostic and therapeutic applications [3].

Imaging and therapeutic strategies in the near-infrared (NIR) region (700–1000 nm) are especially advantageous for in vivo applications due to their enhanced tissue penetration and low absorption in biological tissues [2]. According to Mie-Drude theory, a red shift (the shift in the localized surface plasmon resonance maximum to longer wavelengths) occurs in gold nanoparticles as their size increases and depends on their shape, such as gold nanorods (GNRs), gold nanoprisms (NPRs), and others, regardless of whether they are composed of individual larger particles or agglomerates of smaller ones. In this context, GNRs have been developed with longitudinal plasmon resonance bands located in the NIR region [2]. When functionalized with the D1 peptide (sequence qshyrhispaqv, written in lowercase to indicate the presence of D-configured amino acids), these GNRs enable the detection and disaggregation of Aβ aggregates and reduce their associated toxicity, thereby serving as a promising diagnostic and therapeutic tool for Alzheimer’s disease (AD) [4]. The D configuration offers advantages over the naturally predominant L form, including reduced susceptibility to enzymatic degradation, improved stability, and prolonged biological activity. Incorporating D-amino acids may also enhance receptor binding and modulate biological functions [5]. Additionally, gold nanostars have been conjugated with the D1 peptide to promote Aβ aggregate disaggregation and enable detection via Raman spectroscopy [6]. Moreover, in a prior study focusing on oligomeric Aβ species, considered the most toxic form, we conjugated gold nanorods (NPRs) with the D3 peptide. The all-D enantiomeric peptide D3, composed of the amino acid sequence rprtrlhthrnr, was developed to disrupt and eliminate these cytotoxic Aβ aggregates [7]. D3 was identified through mirror-image phage display screening against monomeric Aβ_42_ from a peptide library comprising over one billion random 12-mer sequences. In vitro studies have demonstrated that D3 selectively eliminates cytotoxic Aβ oligomers by converting them into non-toxic, non-amyloidogenic, and non-fibrillar assemblies. In vivo, D3 has shown therapeutic efficacy across three distinct transgenic mouse models of Alzheimer’s disease. Notably, it reduced amyloid plaque burden and cerebral inflammation following direct intracerebral administration in tg APPswe/PS1ΔE9 mice. Furthermore, D3 inhibited the progression of motor neuron degeneration in HOM TBA2.1 mice after intraperitoneal administration and improved cognitive performance following oral treatment in tg APPswe/PS1ΔE9 mice [7].

D3-conjugated NPRs (gold nanoprisms linked to the D3 peptide) decreased Aβ-induced toxicity in a blood–brain barrier organ-on-a-chip model [8]. Although gold nanorods (GNRs), gold nanoprisms (NPRs), and gold nanostars are highly promising for advancing toward clinical trials, concerns persist regarding their biodegradability, long-term accumulation, and potential toxicity. Therefore, developing biodegradable and excretable nanomaterials is a crucial step toward clinical research. The red shift for the plasmon band is not exclusive to aggregation; it can occur either from increased individual particle size or from agglomeration of nanoparticles, which results in electromagnetic coupling between closely spaced particles.

Unlike NPRs, GNRs, and nanostars, nanosized agglomerates of small gold nanoparticles (sAuNPs) with diameters of about 5–6 nm exhibit enhanced NIR absorption across a broad wavelength range (650–950 nm) due to the red-shift phenomenon. These assemblies also show a strong photothermal effect [9] and, because of their small size, can be excreted in urine through glomerular filtration. In contrast, isolated sAuNPs primarily respond to visible light in the 500–580 nm range, which has limited tissue penetration because of absorption and scattering by hemoglobin, skin, and other tissue components [9]. Isolated sAuNPs (5–6 nm) do not exhibit significant NIR absorption, and only their assembly into agglomerates produces an NIR band via plasmon coupling. Nevertheless, hierarchical assembly of sAuNPs enables tuning of localized surface plasmon resonance (LSPR) peaks into the NIR region, allowing for photothermally controlled release and generating a photothermal effect capable of inducing tumor cell death [10]. After treatment, sAuNPs can disaggregate under biological conditions, aiding their clearance from the body. In this work, sAuNPs were incorporated into albumin nanoparticles to create NIR-absorbing nanomaterials capable of inhibiting Aβ aggregation.

Nanosystems based on bovine serum albumin (BSA) are among the most promising platforms because of their natural origin, non-immunogenicity, and biodegradability, which help reduce tissue buildup and facilitate regulatory approval [11]. BSA is a natural, biocompatible, biodegradable, and non-toxic protein commonly used as a drug carrier. As the most abundant soluble protein in circulation (>60%), it has high solubility (dissolves easily in body fluids), broad pH stability (remains stable across various pH levels), and thermal resistance (withstands heat), making it an effective macromolecular vehicle for drug delivery. Its strong ability to bind various drugs influences their distribution throughout the body and their pharmacological effects, enhancing its role as a versatile therapeutic platform [12].

The integration of small gold nanoparticles (sAuNPs) into BSA-based nanosystems (see above) can significantly enhance biocompatibility (compatibility with the human body) and colloidal stability (ability to stay evenly dispersed in liquid), offer near-infrared (NIR, wavelengths 700–1000 nm) optical properties, and prevent aggregation in biological media. This results in improved circulation time and biodistribution (distribution of the nanoparticles in the body). Moreover, these nanosystems sustain efficient photothermal conversion under NIR irradiation, enabling externally triggered therapies (activated by external light). Their surface can also be easily functionalized (chemically modified) to attach drugs or biomolecules, supporting targeted applications such as delivering therapeutic agents [13,14].

The goal of this study was to develop a degradable nanosystem with NIR absorption properties, based on sAuNPs encapsulated in BSA and conjugated to the D3 peptide, to prevent Aβ aggregation. After the nanosystem undergoes proteolytic degradation in vivo, the sAuNPs can be released, potentially filtered by the kidneys’ glomeruli due to their small size, and subsequently expelled. To our knowledge, this is the first report of a degradable albumin-based nanosystem containing NIR-absorbing sAuNPs designed to inhibit amyloid aggregation. This platform differs from previously reported gold nanoparticle-based anti-amyloid systems [15,16,17,18,19] in that it can be broken down in vivo.

In biological environments such as plasma, the nanosystem must resist premature degradation to maintain therapeutic efficacy for the required duration [20]. However, it should ultimately undergo degradation to allow for safe excretion. Enzymatic degradation of the nanosystem is therefore crucial for this final process. Consequently, an appropriate balance between plasma stability and tissue degradability must be achieved.

To assess the stability of the nanosystem against enzymatic activity, it was incubated with trypsin. Trypsin is an enzyme involved in protein breakdown in the body; it is produced by the pancreas, secreted into the gastrointestinal tract, and is also found in plasma at very low levels. Incubation with this enzyme is a common method for evaluating the degradation of protein-based nanosystems [21]. It enables assessment of biodegradability under low proteolytic activity, helping determine nanosystem stability before reaching the target site. Additionally, using media with high proteolytic activity enables analysis of the nanosystem’s ability to degrade at the target site and its subsequent systemic clearance [22].

**Figure 1:**
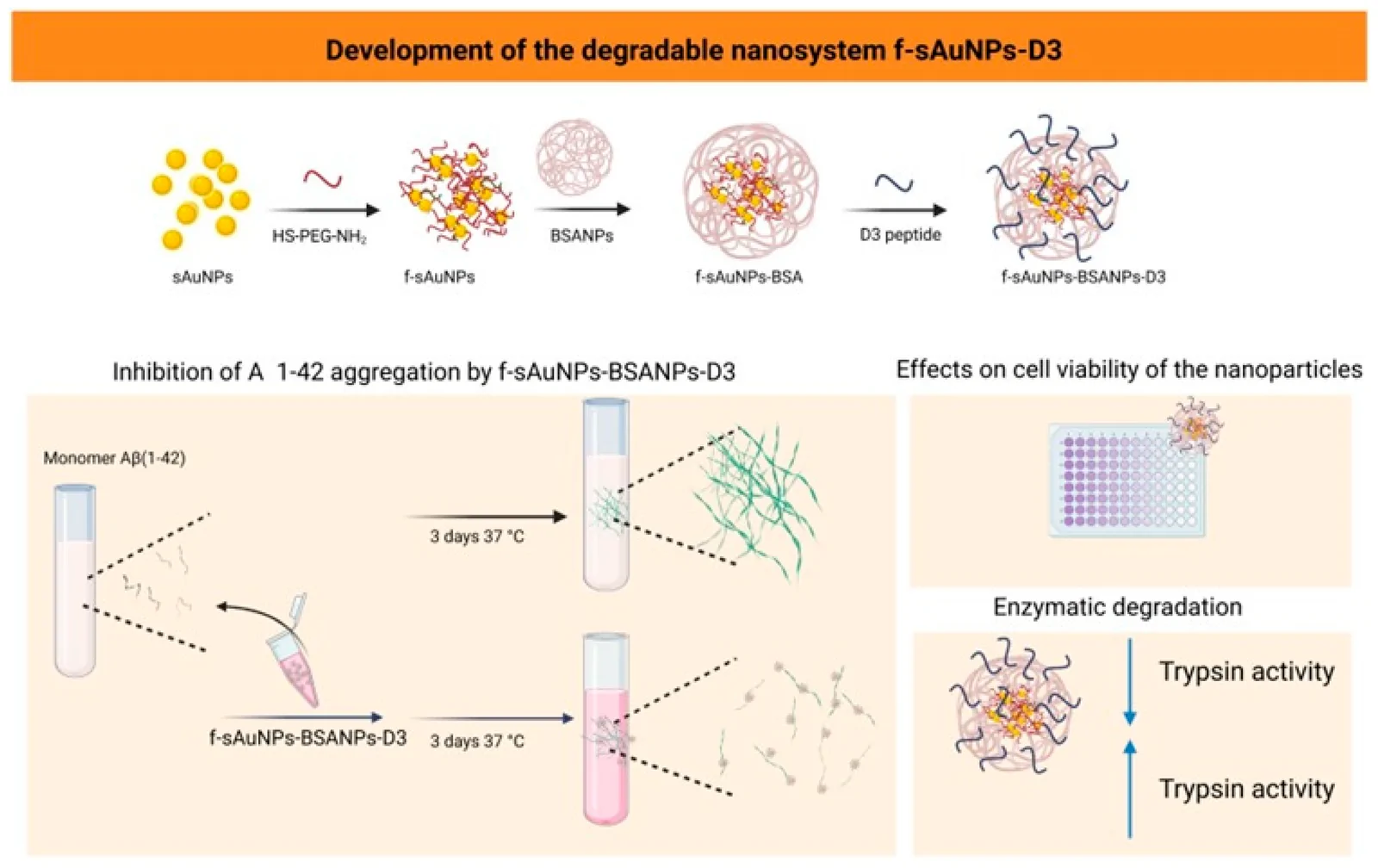
A schematic representation of the preparation of f-sAuNPs-BSANPs-D3 and the evaluation of their effects on Aβ aggregation and neuronal cell viability. The enzymatic degradation of the nanosystem was evaluated in vitro in the presence of trypsin. Licensed by Biorender.

Small gold nanoparticles (sAuNPs) were synthesized using a modified Turkevich method. sAuNPs functionalized with HS-PEG-NH_2_ (f-sAuNPs) were encapsulated into albumin nanoparticles (BSANPs), which were then conjugated with the D3 peptide to produce f-sAuNPs-BSANPs-D3 (Figure 1). The nanosystem was characterized by UV–Vis–NIR spectroscopy, dynamic light scattering, zeta potential measurements, and electron microscopy. Its photothermal properties were evaluated, and its impact on Aβ aggregation was assessed using a thioflavin assay and transmission electron microscopy (TEM). Nanosystem degradation at different trypsin concentrations was measured with dynamic light scattering (DLS). Additionally, the effects on neuronal cell viability were evaluated (Figure 1).

## 2. Materials and Methods

### 2.1. Synthesis of Small Gold Nanoparticles (sAuNPs)

Before nanoparticle synthesis, all glassware and magnetic stir bars were thoroughly cleaned with freshly prepared aqua regia (HCl:HNO_3_, 3:1 *v*/*v*) and extensively rinsed with Milli-Q water (Merck Millipore, Burlington, MA, USA). Small gold nanoparticles (sAuNPs) were synthesized using a modified Turkevich method [10], in which tetrachloroauric acid (HAuCl_4_, Sigma-Aldrich, Saint Louis, MO, USA) was reduced with trisodium citrate (Na_3_C_6_H_5_O_7_, Sigma-Aldrich). To promote gold nucleation, sodium borohydride (NaBH_4_, Sigma-Aldrich) was introduced, enabling synthesis under milder conditions and removing the need for heating. For the synthesis, 50 mL of a 29.4 mM tetrachloroauric acid solution was prepared in a flask and kept at room temperature in ambient air. Next, 2 mL of 38.8 mM sodium citrate was added dropwise while stirring at 600 rpm. After 2 min, 1 mL of sodium borohydride solution (0.075% *w*/*v* in sodium citrate) was added dropwise while stirring until the solution changed from pale yellow to red. The pH was then adjusted to 8 by adding 0.5 M sodium hydroxide (NaOH, Sigma-Aldrich), and the pH change was verified using a pH meter (Jenco^®^, VisionPlus pH6175, San Diego, CA, USA).

#### PEGylation of sAuNPs to Obtain F-sAuNPs

After synthesis, sAuNPs were coated with HS-PEG-NH_2_ (PEG 5000, JenKem Technology, Plano, TX, USA). Briefly, 2 mg of HS-PEG-NH_2_ was dissolved in 100 μL of Milli-Q water (Merck, Simplicity UV, Burlington, MA, USA), then added to the nanoparticle suspension. The mixture was stirred at 600 rpm for 12 h at room temperature.

UV–Vis–NIR absorption spectra and size distribution were recorded at 25 °C using a PerkinElmer Lambda 25 spectrophotometer and a Malvern Zetasizer 3000 (Malvern Instruments, Malvern, UK), respectively. For spectroscopic measurements, 1 mL of the colloidal sample was placed in a quartz cuvette with a 1 cm optical path length. Zeta potential measurements were performed in aqueous solution at a moderate electrolyte concentration using a Zetasizer 3000 (Malvern Instruments, UK), and each measurement was repeated five times. The Smoluchowski approximation was used to calculate zeta potentials from measured electrophoretic mobilities.

Nanoparticle morphology was examined using transmission electron microscopy (STEM) on an FEI Inspect F50 microscope (FEI Company, Waltham, MA, USA) with the following settings: 30 kV accelerating voltage, 500,000× magnification, 597 nm horizontal field width, 4.3 mm working distance, and a spot size of 3.0. For electron microscopy, a drop of the nanoparticle suspension was placed on a Formvar/carbon-coated copper grid (Ted Pella Inc., Redding, CA, USA) and dried at room temperature before imaging.

### 2.2. Synthesis of BSA Nanoparticles (BSANPs)

To synthesize BSANPs, 50 mg of bovine serum albumin (BSA; Sigma-Aldrich) was dissolved in 2 mL of Milli-Q water adjusted to pH 10, and stirred at 500 rpm for 5 min at room temperature. Then, 8 mL of cold absolute ethanol (Sigma-Aldrich) was added dropwise while stirring continuously. After completing the addition, add 12 μL of 0.08% glutaraldehyde (Sigma-Aldrich), and stir the mixture in the dark for 24 h to promote cross-linking (Supplementary Materials, Figures S4–S6).

The nanoparticles were purified through two centrifugation cycles at 25,000× *g* for 1 h at 4 °C using a refrigerated microcentrifuge (LabTech^®^, 1730R, Sorisole, Italy). Each cycle was carried out in 1.5 mL Eppendorf LoBind tubes containing 1 mL of an aliquot. After each centrifugation, the supernatant was discarded, and the pellet was resuspended in Milli-Q water adjusted to pH 10. The final suspension was stored in amber vials at 4 °C until further use [23].

### 2.3. Encapsulation of F-sAuNPs Within BSANPs

For encapsulation, 50 mg of bovine serum albumin (BSA; Sigma-Aldrich) was dissolved in 2 mL of Milli-Q water, adjusted to pH 10 with 0.5 M sodium hydroxide (NaOH), and agitated at 500 rpm for 5 min at room temperature. Subsequently, 3 mL of PEG-functionalized sAuNPs were added, and stirring was continued for an additional 10 min. Cold absolute ethanol (20 mL; Sigma-Aldrich) was then added dropwise while the mixture was continuously agitated. After completion of the addition, 30 μL of 0.08% glutaraldehyde (Sigma-Aldrich) was added, and the mixture was agitated in the dark for 24 h to ensure cross-linking [10].

The nanoparticles were purified through two centrifugation steps at 25,000× *g* for 1 h at 4 °C using a refrigerated microcentrifuge (LabTech^®^, 1730R). Each step was carried out in 1.5 mL Eppendorf LoBind tubes containing 1 mL of an aliquot. After each centrifugation, the supernatant was discarded, and the pellet was resuspended in Milli-Q water adjusted to pH 10 with 0.5 M sodium hydroxide (NaOH). The final suspension was stored in amber vials at 4 °C until further use. The encapsulation efficiency of f-sAuNPs within the f-sAuNPs-BSANPs nanosystem was 85 ± 5% (*n* = 3), as measured by atomic absorption spectroscopy (Supplementary Materials, Table S1).

The nanosystem was characterized by UV–Vis–NIR spectroscopy, DLS, zeta potential measurements, and STEM, following protocols similar to those described for f-sAuNPs (Section PEGylation of sAuNPs to Obtain F-sAuNPs). In addition, cryo-TEM analysis of the f-sAuNPs-BSANPs nanosystem was performed.

For cryo-TEM analysis, 5 μL of each sample was pipetted onto the glow-discharged carbon surface of Lacey Carbon 300-mesh copper grids (Ted Pella, Redding, CA, USA). Cryo-immobilization was performed with a Vitrobot Mark III (FEI Company, Eindhoven, The Netherlands) by plunge-freezing in liquid ethane. Samples were kept at 100% humidity, and excess liquid was automatically blotted with filter paper. Vitrified samples were stored in liquid nitrogen until cryo-electron microscopy analysis. Plunge-frozen grids were transferred to a Tecnai F20 transmission electron microscope (FEI, Eindhoven, The Netherlands) using a cryo-holder (Gatan, Pleasanton, CA, USA). Imaging was done at 200 kV and temperatures between −179 and −170 °C under low-dose conditions. Images were captured at 4096 × 4096 pixels with a CCD Eagle camera (FEI, Eindhoven, The Netherlands).

### 2.4. D3 Peptide Synthesis

For peptide synthesis, 2-chlorotrityl chloride resin, diisopropylcarbodiimide (DIC), OxymaPure, and Fmoc-protected amino acids were purchased from Iris Biotech GmbH (Marktredwitz, Germany). N,N-Dimethylformamide (DMF), methanol (MeOH), dichloromethane (DCM), piperidine, N-ethyldiisopropylamine (DIPEA), trifluoroacetic acid (TFA), and triisopropylsilane (TIS) were obtained from Merck KGaA (Darmstadt, Germany).

Peptide D3 (sequence: rprtrlhthrnr) was synthesized by standard Fmoc solid-phase pep-tide synthesis on 2-chlorotrityl chloride resin (loading: 1.0 meq g^−1^). DIC was used as the coupling agent, OxymaPure as the additive, and in situ neutralization was carried out with DIPEA in DMF. Fmoc deprotection was performed using 20% piperidine in DMF. The peptide was cleaved from the resin using a TFA/TIS/water mixture (95:2.5:2.5, *v*/*v*/*v*) and subsequently purified by chromatography to greater than 98% purity, with an overall synthetic yield of 88%. Molecular mass and purity were confirmed by electrospray ionization mass spectrometry (UPLC Acquity/Xevo G2-XS QToF, Waters Corporation, Milford, MA, USA) (Supplementary Materials, Figure S1A,B).

### 2.5. Conjugation of F-sAuNPs-BSANPs with D3 Peptide to Obtain F-sAuNPs-BSANPs-D3

Before purification, the sample was conjugated with the D3 peptide (sequence: rprtrlhthrnr). The term “D3” indicates the stereochemistry of the peptide, where all amino acids are in the D-configuration. Conjugation was performed as follows: Briefly, 2 mg of EDC (1-ethyl-3-(3-dimethylaminopropyl)carbodiimide; Sigma-Aldrich) was dissolved in 10 mL of Milli-Q water (pH approximately 7; Merck, Simplicity UV). At the same time, 2 mg of NHS (N-hydroxysuccinimide; Sigma-Aldrich) was dissolved in 10 mL of Milli-Q water (pH approximately 7). From these stock solutions, 0.65 mL of EDC and 0.40 mL of NHS were each vortexed for 2 min, then combined and vortexed for an additional 5 min. Next, 1 mg of the D3 peptide was added, and the mixture was vortexed for 5 to 10 min at room temperature.

After activation, 40 µL of the peptide solution was slowly added drop by drop to the f-sAuNPs-BSANPs suspension, with one drop every 5 min. Once the addition was complete, the mixture was vortexed and incubated with gentle agitation at room temperature in the dark for 2 h. The purification process involved centrifugation, removing the supernatant, and resuspending the pellet in Milli-Q water adjusted to pH 10 to remove residual ethanol and unconjugated peptide. The suspension was then shaken for an additional 2 h at ambient temperature and pressure to ensure all ethanol was eliminated before characterization.

#### 2.5.1. Determination of the Conjugation Efficiency of D3 to F-sAuNPs-BSANPs

The conjugation efficiency was indirectly assessed by measuring the amount of free peptide remaining after the reaction between f-sAuNPs-BSANPs and D3. A calibration curve for the D3 peptide was established using four standard concentrations prepared by dissolving known amounts of the peptide in Milli-Q water. Each standard solution was injected into the HPLC system, and the resulting peak areas were recorded to create the calibration curve (Supplementary Materials, Figure S2).

After the conjugation reaction, the nanosystem was centrifuged, and the supernatant (15 mL total volume) containing the non-conjugated peptide was collected. The recovered supernatant was then lyophilized and reconstituted in 1 mL of Milli-Q water before analysis. The concentration of free D3 peptide in the reconstituted sample was measured by HPLC using the previously established calibration curve. Conjugation efficiency was calculated based on the amount of free peptide and the nanoparticle concentration determined by nanoparticle tracking analysis (NTA).

#### 2.5.2. Characterization of F-sAuNPs-BSANPs-D3

At each modification step, nanoparticles were characterized using UV–visible spectrophotometry (Analytik Jena^®^, Specord S600, Jena, Germany) and through dynamic light scattering (DLS) and zeta potential measurements with a Zetasizer Nano ZS (Malvern Panalytical^®^, Malvern, UK). DLS analyses were conducted with folded capillary cells (DTS1070), as described in Section 2.3. f-sAuNPs-BSANPs-D3 were analyzed by nanoparticle tracking analysis (NTA) to determine size distribution and particle concentration with the equipment Nanosight^®^ NS300 (Malvern, UK). The parameters for EV detection included a camera level of 9 and automatic settings for all post-acquisition parameters, except the detection threshold, which was set at 3.

#### 2.5.3. Evaluation of the Effect of NIR Laser Irradiation on F-sAuNPs-BSANPs-D3

A solution of f-sAuNPs-BSANPs-D3 (4.56 nM) was irradiated with an 808 nm laser (350 mW) for 1 h, with the laser positioned 15 cm from the sample. The temperature increase was monitored using a thermal imaging camera (FLIR^®^ E5 Pro, Wilsonville, OR, USA). BSANPs served as a control and were tested under the same conditions. UV–Vis–NIR spectra of the irradiated samples were then recorded.

### 2.6. In Vitro Aβ_1–42_ Aggregation Assay (Fluorescence and TEM Studies)

An aliquot of 0.05 mg of Aβ_1–42_ was dissolved in 300 μL of hexafluoroisopropanol (HFIP) and incubated for 20–30 min at room temperature. The sample was then frozen in liquid nitrogen for 3 min and lyophilized for at least 24 h. The lyophilized peptide was reconstituted in 16 μL of HFIP until fully dissolved, then diluted with 930 μL of ice-cold Milli-Q water, followed by the addition of 945 μL of Milli-Q water. Aliquots of 99 μL were transferred into 250 μL microcentrifuge tubes, and 1 μL of the test compound or nanosystem was added to achieve a final nanosystem concentration of 1 nM. The final concentration of Aβ_1–42_ in the 100 μL assay volume was 11.1 μM. Samples were incubated at 37 °C for 72 h under constant agitation at 300 rpm.

A 1 mM thioflavin T (ThT) stock solution was prepared, diluted to 100 μM, and filtered before use. After incubation, samples were kept on ice, and 20 μL of ThT solution and 80 μL of the corresponding nanosystem were added to each tube. The samples were then transferred into 384-well plates in quadruplicate (50 μL per well) and analyzed with a fluorescence microplate reader.

For TEM analysis, aliquots of Aβ_1–42_ (25 μL), incubated with or without nanoparticles, were placed on Formvar-coated copper grids for 2 min. The grids were then rinsed with Milli-Q water (1 min per rinse) and stained for 2 min with a freshly prepared 1% (*w*/*v*) uranyl acetate solution. After air-drying for 24 h, the samples were examined using a Hitachi HT7700 transmission electron microscope (Hitachi, Tokyo, Japan).

### 2.7. Cell Viability Assays

#### MTS Assay

The SH-SY5Y cell line was obtained from the American Type Culture Collection (ATCC, Manassas, VA, USA; HTB-26TM). The cells were seeded in 96-well plates at a density of 50,000 cells per well and cultured in Dulbecco’s Modified Eagle Medium F12 (DMEM-F12) supplemented with 10% fetal bovine serum (DMEM/FBS). Cells were incubated at 37 °C in a humidified atmosphere containing 5% CO_2_ for 24 h. Serial dilutions (150 µL) of the nanosystem f-sAuNPs, non-functionalized BSANPs, f-sAuNPs-BSANPs, and f-sAuNPs-BSANPs-D3 were prepared in Eppendorf tubes at concentrations of 10, 7, 5, 3, and 1 nM. After removing the culture medium, 90 µL of fresh medium was added to each well. The experimental design tested each nanoparticle formulation and concentration in quadruplicate. Three control conditions were included: DMEM-F12/FBS (10%) as a viability control, Milli-Q water as a vehicle control, and 1% SDS as a cell death control, all in triplicate. Each well received 10 µL of the respective nanosystem dilution or control solution. Plates were incubated for 24 h at 37 °C with 5% CO_2_.

After incubation, the medium was discarded and replaced with fresh medium containing the MTS reagent. Spectrophotometric blanks were prepared in wells without cells, containing 100 µL of medium and MTS reagent. Plates were incubated for 2 h at 37 °C, and absorbance was measured at 490 nm using a Multiskan plate reader (Thermo Fisher, Waltham, MA, USA).

### 2.8. Degradation of F-sAuNPs-BSA-D3 by Trypsin

Proteolytic media with trypsin were prepared in PBS at pH 7.5, with final concentrations of 0.001% (10 µg/mL) to simulate duodenal proteolytic activity, and 0.00001% (0.1 µg/mL) to mimic plasma conditions in a patient without pancreatic pathologies. Each reaction had a total volume of 150 µL and included 25 µL of each nanosystem, resulting in final concentrations of 0.2 nM for BSANPs, f-sAuNPs-BSANPs, and f-sAuNPs-BSANPs-D3 [20,24].

Samples were incubated at 37 °C, and aliquots were taken over five days for DLS analysis. Before these tests, preliminary trials were conducted using trypsin-free PBS samples to find the right dilution factor for DLS measurements, aiming for a correlation function intercept between 0.8 and 0.9 as a starting point.

### 2.9. In Vitro Aβ_1–42_ Aggregation Assay in the Presence of F-sAuNPs-BSA-D3and Irradiation with Near-Infrared Laser (Fluorescence Study)

An aliquot of 0.05 mg of Aβ_1–42_ was dissolved in 300 μL of hexafluoroisopropanol (HFIP) and incubated for 20–30 min at room temperature. The sample was then frozen in liquid nitrogen for 3 min and lyophilized for at least 24 h. The lyophilized peptide was reconstituted in 16 μL of HFIP until fully dissolved, then diluted with 930 μL of ice-cold Milli-Q water, followed by the addition of 945 μL of Milli-Q water. Aliquots of 99 μL were transferred into 250 μL microcentrifuge tubes, and 1 μL of the nanosystem test was added to achieve a final nanosystem concentration of 1 nM. The final concentration of Aβ_1–42_ in the 100 μL assay volume was 11.1 μM. Samples were irradiated with an 808 nm laser (350 mW) for 1 h, then incubated at 37 °C for 72 h under constant agitation at 300 rpm. The laser was positioned 15 cm from the sample.

A 1 mM Thioflavin T (ThT) stock solution was prepared, diluted to 100 μM, and filtered before use. After incubation, samples were kept on ice, and 20 μL of ThT solution along with 80 μL of the corresponding nanosystem were added to each tube. The samples were then dispensed into 384-well plates in quadruplicate (50 μL per well) and analyzed with a fluorescence microplate reader.

## 3. Results and Discussion

### 3.1. Development of the Nanosystem F-sAuNPs-BSANPs-D3

#### 3.1.1. Obtention of F-sAuNPs

sAuNPs were synthesized by reducing gold salt in the presence of sodium borohydride and citrate. The optical properties of the sAuNPs were characterized using UV–Vis spectroscopy, which revealed a strong absorption band centered at 517.5 nm (Supplementary Materials, Figure S3A). This band corresponds to the localized surface plasmon resonance (LSPR) of spherical gold nanoparticles with diameters of approximately 5–6 nm, consistent with previously reported data [25].

**Figure 2:**
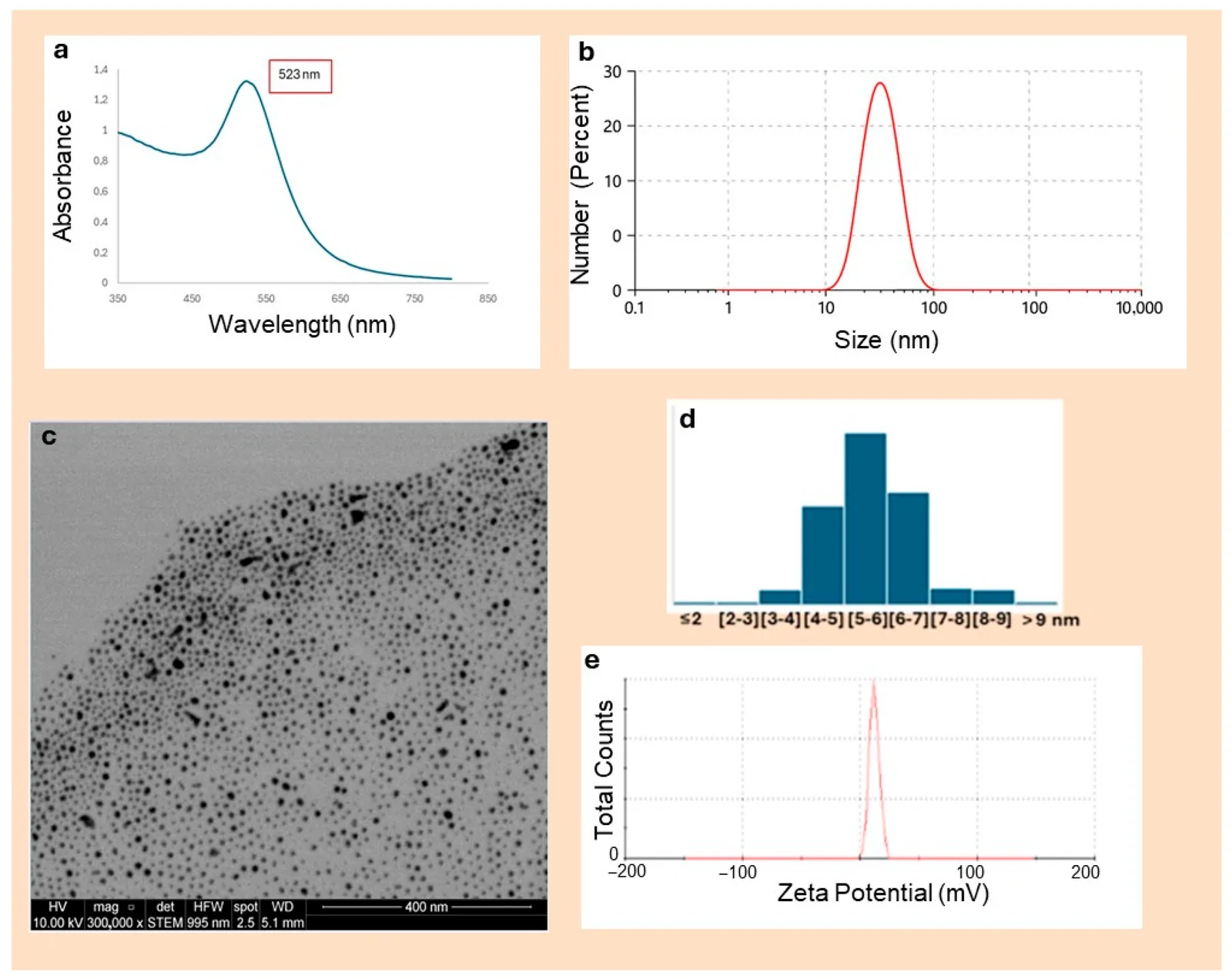
Characterization of f-sAuNPs. (**a**) A UV–Vis–NIR absorption spectrum showing a surface plasmon resonance band at about 523 nm. (**b**) Intensity-weighted size distribution obtained by dynamic light scattering (DLS). (**c**) A STEM micrograph displaying mainly spherical nanoparticles with individual diameters in the nanometer range. Scale bar: 400 nm. (**d**) Size distribution of f-sAuNPs. (**e**) The zeta potential (ζ) profile.

After functionalization with HS-PEG-NH_2_, a slight red shift in the LSPR band (from 517.5 nm to 523 nm) was observed (Figure 2a), indicating surface modification of the gold nanoparticles due to changes in the local dielectric environment. The sharpness and well-defined position of the absorption peak suggest a well-dispersed nanoparticle population in the nanometer range, with minimal aggregation, as aggregation would typically cause a redshift and broadening of the peak. These results confirm the production of homogeneous sAuNPs [26].

Dynamic light scattering (DLS) analysis indicated a hydrodynamic diameter of 35 ± 10 nm (PDI = 0.255) (Figure 2b), while scanning transmission electron microscopy (STEM) showed a uniform population with an average core diameter of 5.7 ± 1.1 nm (range approximately 4–11 nm) (Figure 2c,d). The observed polydispersity is typical for chemically synthesized metal nanoparticles and does not adversely affect their optical properties or suitability for biomedical applications [27,28]. No large aggregates were seen, suggesting that HS-PEG-NH_2_ functionalization effectively maintains colloidal stability and prevents particle coalescence.

Functionalized sAuNPs (f-sAuNPs) exhibited a positive zeta potential of +10 ± 1 mV (Figure 2e), which can be attributed to partial protonation of the terminal amino groups at pH 8. This behavior agrees with previous reports by Wei et al. [29], who showed that HS-PEG-NH_2_ functionalization shifts the zeta potential of AuNPs toward positive values without significantly affecting colloidal stability.

#### 3.1.2. Encapsulation of F-sAuNPs in Albumin Nanoparticles

The nanosystem f-sAuNPs-BSANPs was formed via albumin desolvation in the presence of sAuNPs, following the method outlined by Park et al. [10]. The primary interaction between the positively charged f-sAuNPs (zeta potential of about +10 mV) and negatively charged BSA (isoelectric point around 4.7) is mainly due to electrostatic forces.

Nanoparticles based on bovine serum albumin (BSA), prepared through the desolvation method, have proven to be highly versatile and reproducible drug delivery systems [30,31]. It has been shown that controlling key synthesis parameters, such as pH and the cross-linking agent, allows for the creation of particles with narrow size distributions. Standardized BSA protocols generally produce particle sizes of 100–300 nm, which are considered ideal for colloidal stability and drug loading [30]. Furthermore, BSA’s resistance to organic solvents and thermal changes ensures high reproducibility in manufacturing these nanocarriers.

**Figure 3:**
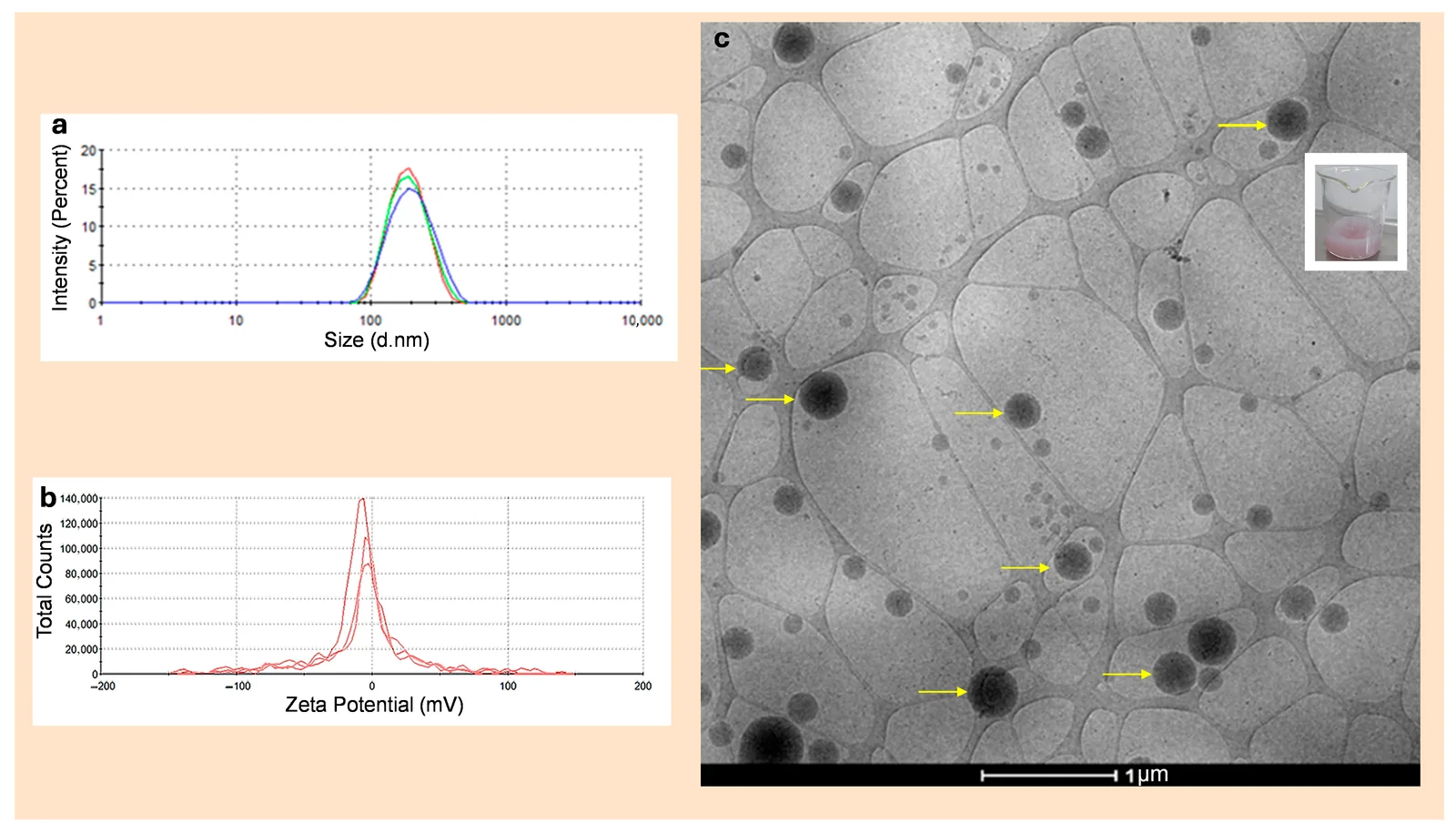
Physicochemical and morphological characterization of the sAuNPs-BSANPs nanosystem. (**a**) DLS size distribution showing a single, well-defined peak at 209 nm across three replicates, indicating a homogeneous particle population with no detectable aggregation. In red, green, and blue are shown the three measurements of the same sample. (**b**) Zeta potential distribution with the main population close to neutrality (−0.15 to −7.13 mV, representing 66–77% of the particles) and minor subpopulations exhibiting more pronounced negative or less common positive surface charges, characteristic of protein-based nanosystems stabilized mainly by steric effects. (**c**) A cryo-TEM micrograph of the hydrated nanosystem, showing electron-dense sAuNPs evenly distributed within the BSA matrix (yellow arrows indicate individual sAuNPs). The inset shows an aqueous dispersion of the sAuNPs-BSANPs-D3 nanosystem.

DLS characterization of the sAuNPs-BSANPs showed unimodal intensity distributions across all three measurements (Figure 3a). The average hydrodynamic diameter was approximately 209 nm. The polydispersity index (PDI) remained low across all replicates (0.163 ± 0.019), indicating a narrow, well-defined colloidal population. In each case, a single peak accounting for 100% of the intensity was observed, with no signs of secondary populations or aggregates in the micrometric range. Overall, these results confirm that the formulation is uniform and maintains good colloidal stability under the tested conditions.

Electrophoretic characterization by zeta potential measurements revealed that sAuNPs-BSANPs mainly showed a distribution centered around neutrality, with minor subpopulations exhibiting more negative charges (Figure 3b). The average zeta potential across three replicates was −10.7 ± 0.8 mV, aligning with systems stabilized mainly by steric rather than electrostatic effects. Each measurement displayed three peaks: the primary peak, accounting for 66–77% of the population, was between −0.15 and −7.13 mV, while secondary peaks, contributing only 4–11%, ranged from −40 to −65 mV or occasionally to small positive values. This heterogeneity is typical of protein-based nanoparticles, where electrophoretic mobility reflects differences in conformation or surface charge density within the same formulation.

This profile corresponds to what are called “soft” protein nanoparticles, where the typical interpretation of zeta potential may lose its significance because the electrical double layer and electrophoretic mobility are not characterized by a sharp surface but by a diffuse and permeable macromolecular layer [32]. In these systems, colloidal stability is often more affected by the hydrated protein corona and steric interactions than solely by electrostatic repulsion, which explains why low zeta potential values can still be associated with stable dispersions that have heterogeneous surface charges [33,34].

The nanosystem appeared as a pink suspension, indicating the successful incorporation of f-sAuNPs into the albumin nanoparticles (insert Figure 3c). The efficiency of nanoparticle encapsulation in the albumin nanosystem is 87 ± 5% (*n* = 3), as determined by atomic absorption (Supplementary Materials, Table S1). Cryo-TEM analysis (Figure 3c) provided further insight into the hydrated morphology of the nanosystem. Cryo-TEM is a technique that preserves the sample’s native ultrastructure. Nanoparticles approximately 212 nm in diameter were observed for f-sAuNPs-BSANPs, aligning well with the hydrodynamic sizes obtained by DLS (Figure 3a). Electron-dense sAuNPs were clearly encapsulated within the BSA matrix and evenly dispersed throughout the nanosystem. Yellow arrows indicate the positions of individual sAuNPs, highlighting their location inside the protein carrier and supporting the structural stability of the encapsulated nanoparticles in aqueous conditions. Similar results have been reported for hybrid systems where a gold core is encapsulated within albumin-based nanoparticles, such as Au@HSANPs, where TEM images show a uniform metallic core embedded in the protein matrix, demonstrating the structural integrity of these hybrid nanoparticles under biological conditions [35].

#### 3.1.3. Conjugation of F-sAuNPs-BSANPs to the D3 Peptide to Obtain F-sAuNPs-BSANPs-D3

**Figure 4:**
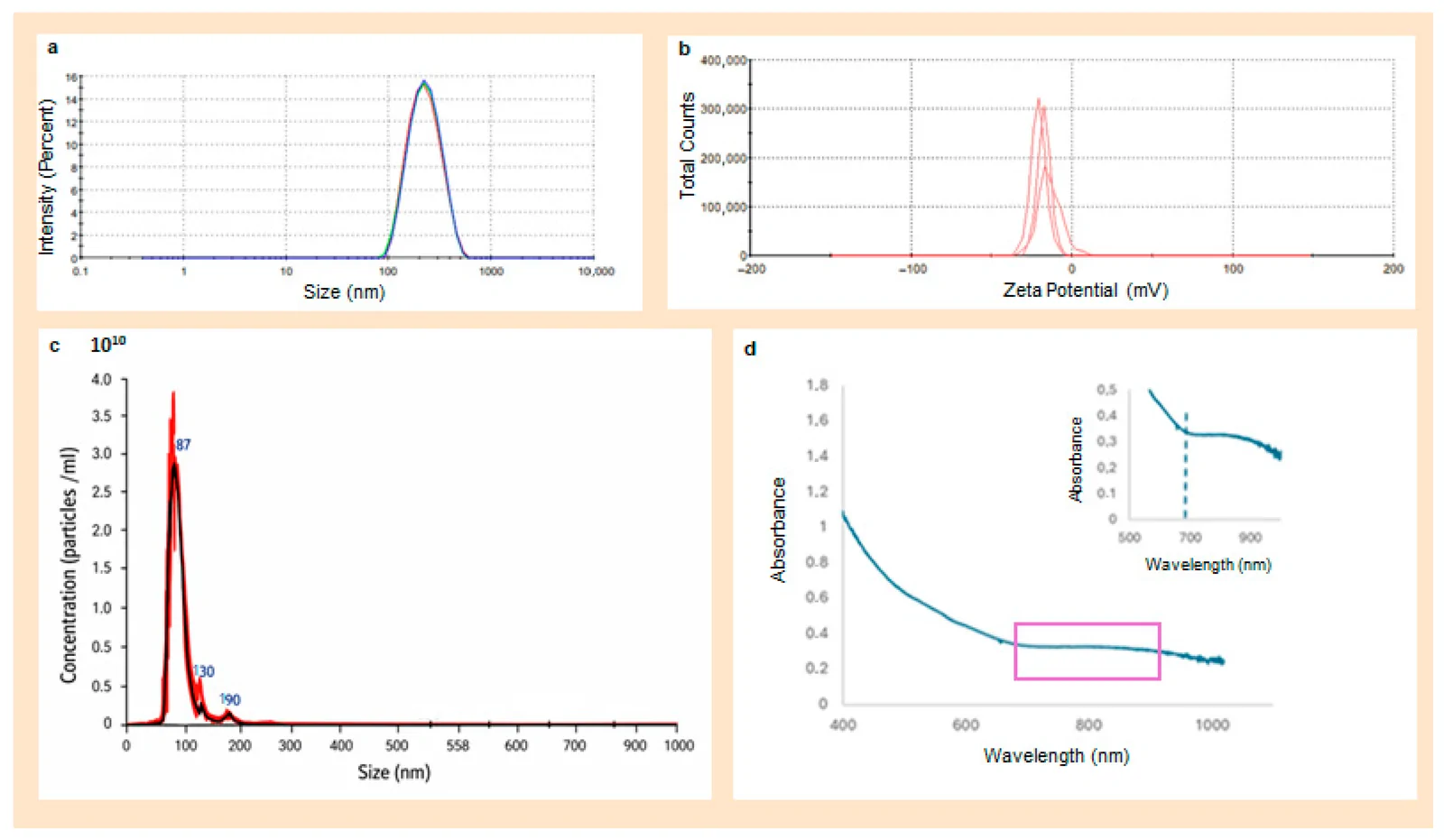
Physicochemical characterization of f-sAuNPs-BSANPs-D3. (**a**) DLS hydrodynamic size distribution by intensity of the f-sAuNPs-BSANPs nanosystem, showing a dominant and consistent population across three independent measurements, indicating a uniform particle population. In red, green, and blue are shown the three measurements of the same sample. (**b**) Zeta potential distribution obtained through electrophoretic analysis, displaying a unimodal profile with similar values among replicates, characteristic of protein-based nanosystems mainly stabilized by steric interactions. (**c**) NTA size distribution of f-sAuNPs-BSANPs. In red and black are shown two measurements of the same sample. (**d**) The UV–Vis-NIR absorption spectrum of f-sAuNPs-BSANPs, revealing a broad plasmonic feature spanning approximately 700–1000 nm. The purple rectangle highlights a region of interest, shown in more detail in the inset (top right), corresponding to plasmon coupling effects in f-sAuNPs-BSANPs-D3.

f-sAuNPs-BSANPs were conjugated with the peptide D3 to form the nanosystem sAuNPs-BSANPs-D3. DLS analysis showed mostly unimodal intensity distributions across all three measurements (Figure 4a). The average hydrodynamic diameter, as determined by cumulant analysis (Z-average), was 233 nm. The polydispersity index (PDI) remained low across all replicates (0.127), indicating a narrow and well-defined colloidal population. All measurements showed a single distribution peak, accounting for 100% of the intensity, which suggests no presence of distinct subpopulations or aggregates with different electrophoretic behaviors. The negative zeta potential values gradually increased (Figure 4b), ranging from −14 to −17 ± 3 mV, possibly due to minor changes in surface conformation or increased exposure of charged functional groups. Notably, all values are within the range typically considered moderately stable for protein-based nanosystems in aqueous suspension. Overall, these results confirm that the f-sAuNPs-BSANPs-D3 formulation maintains a consistent, unimodal surface charge distribution without signs of significant aggregation or subpopulation formation under the tested conditions. The shift toward more negative zeta potential values likely relates to increased exposure of protein-associated charged groups or subtle conformational changes at the nanosystem surface after peptide conjugation—a common indicator of successful surface functionalization in protein nanoparticles. Collectively, these findings show that D3 functionalization does not impair colloidal stability and results in a more homogeneous, moderately charged surface, aligning with values typically reported for protein-based nanosystems in water [36,37].

Characterization through nanoparticle tracking analysis (NTA) enabled the assessment of size distribution based on particle count and concentration of the nanosystem (Figure 4c). The nanosystem exhibited an average particle size of 96 nm and a concentration of 9.16 × 10^10^ particles/mL (Figure 4c). The amount of D3 peptide conjugated to the BSA nanoparticles was measured using a previously established calibration curve from the supernatant obtained after centrifugation-based purification. The conjugation efficiency was estimated by measuring the free peptide in the supernatant with the calibration curve (Figure S2), resulting in an efficiency of 88%. The UV–Vis absorption spectrum of f-sAuNPs-BSANPs (Figure 4d) displayed a broad band from approximately 700 to 1000 nm, which is attributed to plasmon coupling effects caused by sAuNP aggregates confined within the BSA nanoparticles, as previously reported [10,38]. Importantly, the BSANPs do not show any absorption band in the 700–900 nm range (Supplementary Materials, Figure S3B).

### 3.2. Effect of Near-Infrared Irradiation on F-sAuNPs-BSANPs-D3

**Figure 5:**
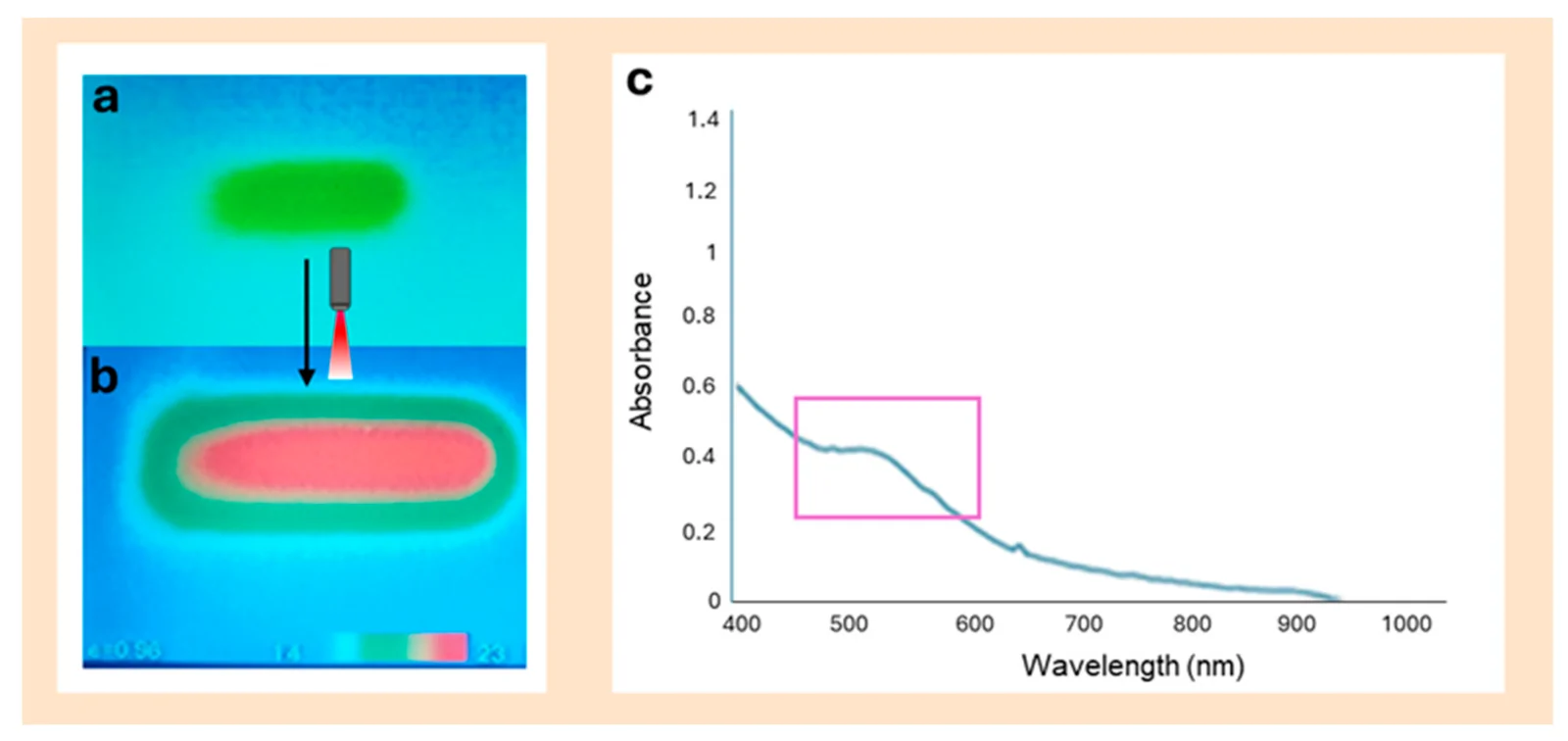
Photothermal response and characterization of f-sAuNPs-BSANPs-D3 after irradiation. (**a**) A thermal image of an Eppendorf vial containing the f-sAuNPs-BSANPs-D3 nanosystem before near-infrared (NIR) irradiation, serving as a visual control prior to photothermal activation. (**b**) A thermal image of the vial after 1 h of irradiation with an 808 nm NIR laser, showing a localized temperature rise due to photothermal conversion by the nanosystem. (**c**) The UV–Vis absorption spectrum of the nanosystem after irradiation, illustrating attenuation of the near-infrared absorption. The purple rectangle highlights a weak residual plasmonic feature attributed to sAuNPs that persists after irradiation, indicating changes in the nanosystem’s structural and optical environment.

The nanosystem f-sAuNPs-BSANPs-D3 was irradiated with an 808 nm laser (power: 350 mW), resulting in a temperature increase of 5 °C (Figure 5a before irradiation and Figure 5b after irradiation, Table S2, Supplementary Materials). The results show that the nanosystem exhibits a photothermal response that depends on its composition, with the presence of sAuNPs encapsulated within the albumin protein matrix being crucial for converting NIR radiation into heat (Supplementary Materials, Table S2). Notably, during NIR irradiation, BSANPs without encapsulated sAuNPs did not display any significant temperature change, whereas irradiating the gold-containing nanosystem caused an approximate temperature rise of 5 °C (Supplementary Materials, Table S2). These findings clearly demonstrate that sAuNPs are essential for the photothermal effect and confirm their key role in enabling photothermal applications.

Consistent with previous studies, it has been shown that sAuNPs (~5 nm) coated with bovine serum albumin (BSA) can produce detectable temperature increases under NIR laser irradiation (~800 nm), supporting the suitability of this system for photothermal applications in biomedicine [10]. The temperature increase observed in the f-sAuNPs-BSANPs-D3 nanosystem can be attributed to the confinement and controlled aggregation of sAuNPs within the protein matrix. When sAuNPs are positioned close together, coupling of their localized surface plasmon resonance (LSPR) modes is enhanced. This effect has been extensively described and is associated with shorter interparticle distances, resulting in a red shift of the LSPR bands [36,37,38]. In fact, biopolymer matrices that trap sAuNPs can influence LSPR by changing local polarizability and interparticle spacing [39]. In this context, BSA acts as a nanoscale scaffold that keeps interparticle distances suitable for controlled plasmonic coupling, enabling effective photothermal responses under NIR irradiation, even for ultrasmall nanoparticles [39].

The UV–Vis–NIR spectrum of the nanosystem f-sAuNPs-BSANPs-D3 after irradiation is shown in Figure 5c. Spectrophotometric analysis indicated that, before irradiation, the nanosystem maintained its characteristic spectral profile. After irradiation, a distinct peak appeared around 500 nm, which is typical of free sAuNPs in solution. This suggests that the laser-induced temperature increase may partially denature the nanosystem, releasing gold nanoparticles into the surrounding medium, as shown in Figure 5c. Comparing the UV–Vis-NIR spectra before and after NIR irradiation (Figure 4d vs. Figure 5c) reveals significant changes in the nanostructural organization of the system. Relative to the non-irradiated sample (Figure 4d), the irradiated nanosystem shows an overall decrease in absorbance throughout the spectral range, along with a reduced localized surface plasmon resonance (LSPR) band in the NIR region. Additionally, the appearance of a well-defined band at approximately 500 nm after irradiation aligns with the loss of interparticle plasmonic coupling due to the confinement of sAuNPs within the BSA matrix, indicating structural reorganization and/or partial release of the metallic cores.

Overall, these spectral changes indicate a modification of the local dielectric environment and/or a reorganization of the nanosystem caused by irradiation, without evidence of complete degradation of the metallic component. Recent studies have shown that NIR irradiation in sAuNP–protein nanosystems can induce conformational changes in the protein coating and alter the local dielectric environment, thereby adjusting the plasmonic response without full protein denaturation [38,39]. Consistent with these findings, the current literature suggests that photothermal efficiency and structural stability heavily depend on nanoparticle confinement and interparticle spacing within organic matrices, supporting the proposed mechanism for this hybrid system.

Although the measured bulk temperature increase in our system under NIR irradiation was modest (~5 °C), this value aligns well with previous reports on plasmonic nanosystems for β-amyloid modulation. Several studies have shown that temperature rises of 5–15 °C are enough to disrupt or inhibit Aβ fibril formation under NIR irradiation, especially when using gold nanoparticles or nanorods [15,16,17,18]. Importantly, plasmonic nanoparticles produce highly localized nanoscale heating that can greatly exceed the measured bulk temperature, effectively destabilizing β-sheet structures [19]. In this context, even moderate increases in overall temperature, such as those observed in our system, are sufficient to induce structural changes in amyloid aggregates. Hence, our results support the significance of the photothermal effect, even under mild irradiation conditions.

### 3.3. Evaluation of the Effects of the Nanosystems on Cell Viability

**Figure 6:**
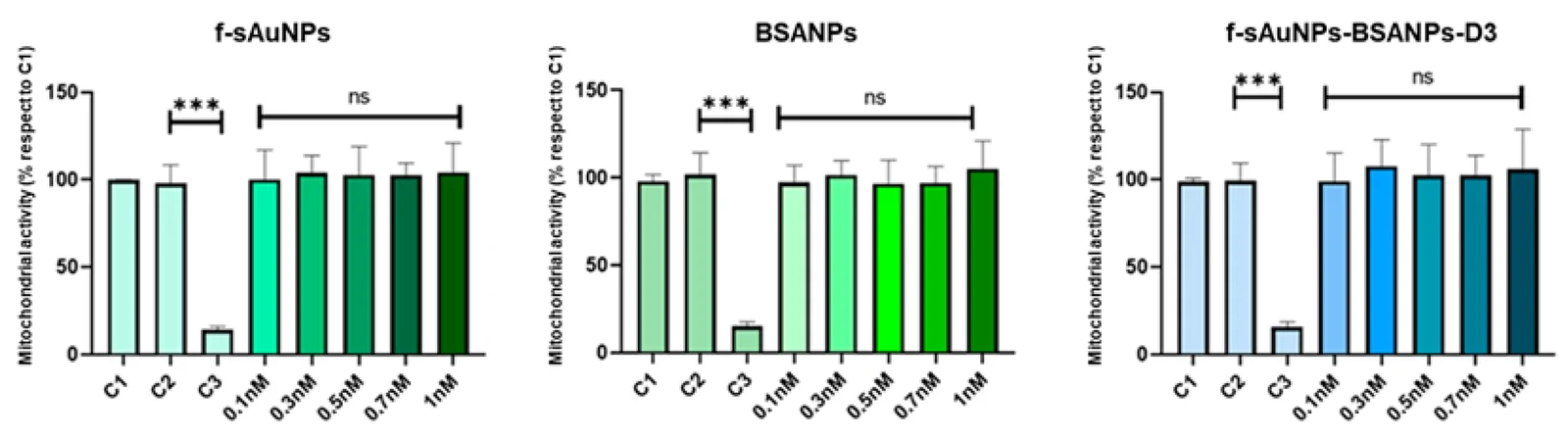
The effects of nanoparticle treatment on cell viability. Cell viability was measured using the MTS assay in SH-SY5Y neuroblastoma cells exposed to various concentrations of the nanosystems. C1: live control; C2: vehicle control; and C3: death control. Experiments were conducted in triplicate (*n* = 3). Cells were treated with f-sAuNPs, BSANPs, and f-sAuNPs–BSANPs–D3 and incubated for 24 h at 37 °C. After treatment, the MTS reagent was added, and cells were incubated for an additional 2 h at the same temperature before measuring cell viability (*** *p* < 0.001, ns: non-significant).

For future use of the nanosystem as a potential treatment for Alzheimer’s disease, it is essential to evaluate its effects on relevant brain cells. Cell viability assays on SH-SY5Y neurons showed that neither the individual components of the nanosystem nor the full formulation negatively affected mitochondrial activity at concentrations between 0.1 and 1 nM. BSANPs, sAuNPs, and f-sAuNPs-BSANPs-D3 all exhibited viability levels similar to the live control, with no significant differences compared to the vehicle control and clearly distinct from the dead control, indicating no cytotoxicity after 24 h of exposure (Figure 6).

BSANPs demonstrated the expected biocompatibility, consistent with the widespread use of albumin as a safe matrix in biomedical applications. Similarly, sAuNPs caused minimal effects on mitochondrial activity, indicating that PEGylation reduces potentially harmful interactions with neuronal cells. Overall, the entire nanosystem did not impact SH-SY5Y cell viability, showing that encapsulating sAuNPs within BSA nanoparticles and functionalizing them with the D3 peptide ensures an adequate safety profile in this neuronal model.

Taken together, these results show that the evaluated formulations have good cellular tolerability, supporting their further exploration for therapeutic use in Alzheimer’s disease, where neuronal biocompatibility is essential [40,41].

### 3.4. Degradation of the F-sAuNPs-BSANPs-D3 Nanosystem

**Figure 7:**
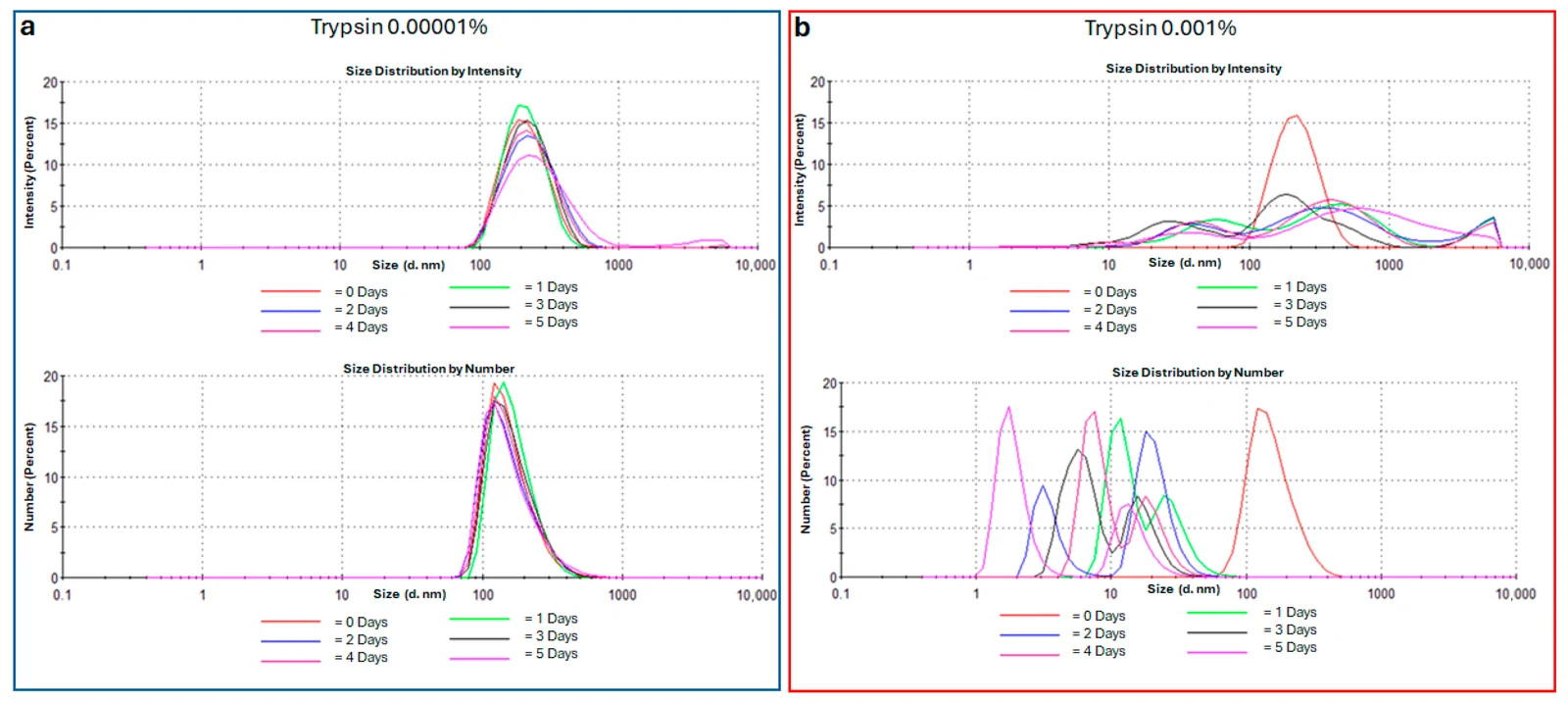
The effect of trypsin on the size distribution of the f-sAuNPs-BSANPs-D3 nanosystem, displayed as intensity (**top**) and number (**bottom**), measured by DLS. The nanosystems were incubated with trypsin at concentrations of 0.00001% (**a**) and 0.001% (**b**) to mimic systemic proteolytic conditions and high protease activity in different compartments, respectively. Samples were maintained at 37 °C in PBS buffer (pH 7.4).

The f-sAuNPs-BSANPs-D3 nanosystem was incubated with trypsin at two different concentrations to examine its degradation behavior under conditions similar to various biological environments. The lower trypsin concentration (0.00001%) was selected to simulate systemic exposure, as observed in plasma (Figure 7a). In contrast, the higher concentration (0.001%) represents proteolytic activity in tissues such as the small intestine (Figure 7b) [42,43].

Analysis of hydrodynamic size and size distribution revealed the different behavior of the nanosystem depending on trypsin concentration. At low trypsin levels (0.00001%), the average hydrodynamic diameter from intensity-weighted measurements remained stable over time, indicating that the nanosystem’s integrity was maintained (Figure 7a) [24]. In contrast, at high trypsin concentrations (0.001%), a broader size distribution was observed, with both larger and smaller particle populations relative to the initial size, indicating nanosystem degradation and reorganization (Figure 7b) [21,24].

Consistently, at 0.00001% trypsin, the number-weighted size distribution remained unchanged, indicating no significant fragmentation processes (Figure 7a). However, at 0.001% trypsin, the number distribution clearly shifted toward smaller diameters, showing that degradation and fragmentation became the main processes (Figure 7b). This behavior results from the formation of small protein fragments through enzymatic hydrolysis of the albumin matrix, along with the release of sAuNPs. These effects are partly masked in the intensity-weighted distributions by scattering from larger aggregates.

The evolution of the correlograms further reinforced these findings. Under conditions of low proteolytic activity, the correlation curves exhibited a monoexponential decay pattern and high correlation intensity, consistent with colloidal stability. However, a slight and reproducible decrease in decay time was noted (Supplementary Materials, Figure S7). In contrast, at 0.001% trypsin, there was a significant reduction in correlation intensity, a shorter decay time, and distortion of the correlogram, indicating the presence of highly mobile small fragments and heterogeneous aggregates.

Overall, these results show that f-sAuNPs-BSANPs-D3 maintain their colloidal stability in conditions like plasma that mimic a low-activity systemic proteolytic environment, thus preserving their structural integrity over time [42,43]. This is especially important when considering the expected plasma half-life of the nanosystem. Conversely, in environments with high proteolytic activity or pathological states [21], the nanosystem undergoes controlled degradation, evidenced by the fragmentation of the albumin matrix, release of albumin, formation of albumin aggregates, and release of sAuNPs.

These findings are consistent with previous reports in the literature. Studies summarized in [39] emphasize the physicochemical strength of albumin, its ability to form stable nanoparticles through methods like desolvation, and its effectiveness in prolonging drug half-life, which aligns with our observation that albumin-based nanosystems remain stable under low proteolytic activity. Conversely, other studies [44,45,46] indicate that BSANPs are susceptible to trypsin-mediated degradation, reporting faster siRNA release and a loss of matrix integrity. This closely resembles the degradation behavior we observed in our system at high trypsin concentrations (0.001%).

Finally, it is important to emphasize the key role of dynamic light scattering (DLS) in evaluating the stability and biodegradability of advanced nanosystems, especially as nanomaterials designed for controlled degradation in biological environments continue to develop [47]. In addition to its use in initial physicochemical characterization—such as optimizing human serum albumin nanoparticles for paclitaxel delivery [48,49]—DLS enables real-time monitoring of dynamic processes, including progressive fragmentation, heterogeneous aggregation, and component release under various proteolytic conditions.

### 3.5. Evaluation of the Effect of F-sAuNPs-BSANPs-D3 on Aβ_1–42_ Aggregation

**Figure 8:**
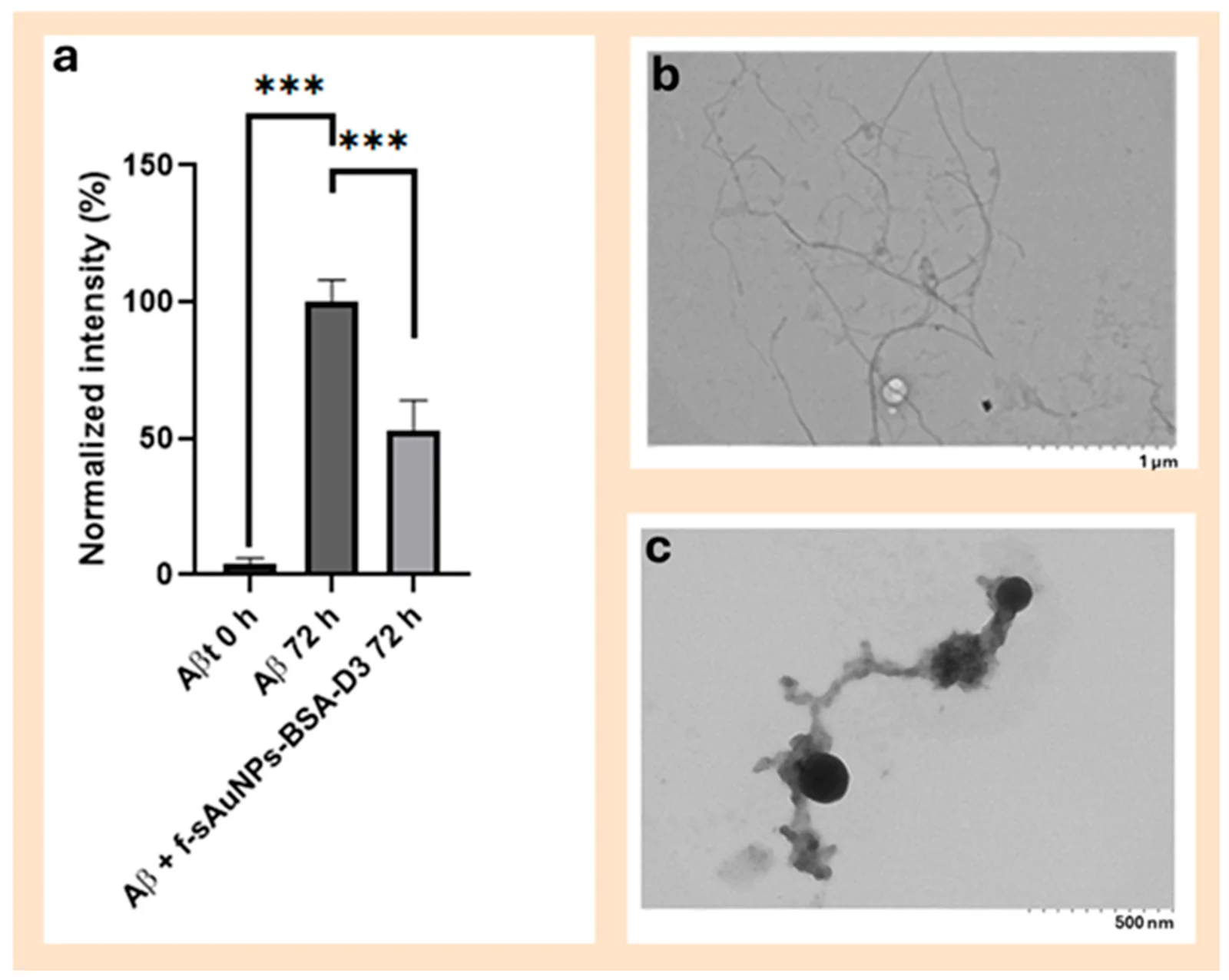
The effect of sAuNPs-BSA-D3 on Aβ_1–42_ aggregation and fibrillar structure. (**a**) The fluorescence intensity of Aβ_1–42_ measured at time zero after 72 h of incubation without nanoparticles, showing a time-dependent increase associated with fibril formation, and in the presence of sAuNPs-BSA-D3 (37 °C, 300 rpm), indicating a significant decrease in fluorescence in the nanoparticle-treated sample. *** *p* < 0.001 (*n* = 3). (**b**) A transmission electron microscopy image of Aβ_1–42_ fibrils incubated at 37 °C and 300 rpm for 72 h. (**c**) A transmission electron microscopy image of Aβ_1–42_ incubated with f-sAuNPs-BSA-D3 at 37 °C and 300 rpm for 72 h, showing a disrupted and less organized fibrillar network.

The Aβ_1–42_ peptide was treated with hexafluoroisopropanol (HFIP) to disaggregate it. Incubating the Aβ_1–42_ peptide for 72 h at 37 °C caused aggregation, demonstrated by a significant increase in Thioflavin T (ThT) fluorescence when comparing unincubated samples (t = 0 h) with those incubated for 72 h (t = 72 h). As shown in Figure 8a, mean fluorescence values increased, confirming the formation of amyloid structures. This pattern aligns with the typical kinetics of Aβ_1–42_ aggregation, where longer incubation promotes the transition from monomeric and oligomeric species to highly ordered fibrils with a strong affinity for ThT [50].

In contrast, incubating Aβ_1–42_ with f-sAuNPs-BSANPs-D3 led to a substantial reduction in ThT fluorescence compared to the peptide alone (Figure 8a). This indicates partial inhibition of aggregation and fewer β-sheet-rich structures recognized by ThT. These results suggest that the nanosystem affects peptide aggregation, potentially by stabilizing less structured species or disrupting fibrillar elongation. Similar effects have been observed with nanostructured systems and D-enantiomeric peptides, which can interfere with Aβ fibril nucleation and growth, redirecting the aggregation pathway toward alternative, less organized states [51].

Consistently, transmission electron microscopy (TEM) analysis revealed that Aβ_1–42_ incubated without nanoparticles formed typical amyloid fibrils, including individual fibers and dense aggregates of mature fibrils (Figure 8b). In contrast, samples incubated with f-sAuNPs-BSANPs-D3 showed a markedly different morphology, characterized by a disrupted, poorly ordered fibrillar network and a predominance of spherical structures arranged in linear formations, similar in size to the nanosystem (Figure 8c). This morphological change supports the ThT fluorescence data. It suggests that the nanosystem interferes with the nucleation and growth of Aβ fibrils, redirecting the aggregation toward smaller, potentially less toxic assemblies. Previous studies have demonstrated that D3 selectively binds to Aβ aggregates and alters their supramolecular organization, aligning with the structural modifications and partial inhibition of aggregation observed in this system [7].

Collectively, these results demonstrate that the nanosystem not only reduces the formation of mature amyloid fibrils but also redirects Aβ aggregation toward different structures, supporting its potential as an Aβ aggregation modulator.

Furthermore, we evaluated the effect on the Aβ_1–42_ aggregation process in the presence of the nanosystem f-sAuNPs-BSANPs-D3 and near-infrared irradiation. Incubating Aβ_1–42_ with f-sAuNPs-BSANPs-D3 and applying irradiation resulted in a significant reduction in ThT fluorescence compared to the non-irradiated nanosystem, indicating that the irradiation process inhibited Aβ_1–42_ aggregation (Supplementary Materials, Figure S8).

## 4. Conclusions

In this study, the nanosystem f-sAuNPs-BSANPs-D3 was developed for potential biomedical applications. f-sAuNPs were integrated into BSANPs under optimized encapsulation conditions, producing stable, homogeneous nanoparticles with a suitable surface charge for biomedical use. Additionally, the D3 peptide was conjugated to inhibit beta amyloid aggregation. The optimized nanosystem demonstrated a notable photothermal response under NIR irradiation while exerting minimal effects on cell viability at the tested concentrations (0.1 nM–1 nM). It also effectively reduced amyloid-β fibril formation, as shown by TEM and thioflavin T fluorescence assays, with around a 50% reduction in fluorescence intensity. Furthermore, NIR irradiation of the nanosystem disrupted the aggregation process.

Proteolytic degradation studies further showed a trypsin concentration-dependent response of the nanosystem. At low protease activity (0.00001%), which roughly simulates physiological plasma conditions, the albumin-based formulation maintained its colloidal stability and structural integrity over time. In contrast, higher trypsin concentrations (0.001%) led to controlled degradation of the albumin matrix, as indicated by the formation of small protein fragments and heterogeneous aggregates and the release of sAuNPs. Overall, these results demonstrate that the nanosystem combines physiological stability with enzyme-triggered biodegradability, a key feature for biomedical applications that need both prolonged circulation and safe degradation. Unlike other gold nanoparticle nanosystems with NIR plasmonic properties, our nanosystem could be degraded in vivo, a feature that warrants exploration in future studies.

## Data Availability

The original contributions presented in this study are included in the article/Supplementary Material. Further inquiries can be directed to the corresponding authors.

## Supplementary Materials

The following supporting information can be downloaded at: https://www.mdpi.com/article/10.3390/pharmaceutics18040504/s1, Figure S1: Characterization of the peptide D3. (A) HPLC analysis of the obtained peptide D3. Gradient water-acetonitrile (0–100%, 15 min), flux 1 ml/min. Retention time 5.7 min. Purity obtained: 97%; (B) Mass spectrum of the peptide D3: ionic fragments; Figure S2: Calibration curve for peptide D3; Figure S3: (A) UV-Vis-NIR spectrum of sAuNP; (B) UV-Vis-NIR spectrum of BSANPs; Figure S4: Size distribution of BSANPs (DLS) obtained by the desolvation method; Figure S5: Zeta potential of BSANPs obtained by the desolvation method; Figure S6: STEM image of BSANPs obtained by the desolvation method; Table S1: Determination of the efficiency of encapsulation of gold nanoparticles in the nanosystem f-sAuNPs-BSANPs; Table S2: Initial temperature, final temperature, and temperature increase in BSANPs and f-sAuNPs-BSA-D3 after 1 h of 808 nm NIR laser irradiation; Figure S7: Effect of trypsin on f-sAuNPs-BSANPs-D3 nanosystem on correlograms obtained by DLS. Nanosystems incubated with 0.00001 (a) and 0.001% (b) of trypsin concentrations to simulate systemic proteolytic conditions and high-activity compartments, respectively. The samples were incubated at 37 °C in PBS buffer at pH 7.4; Figure S8: Effect of sAuNPs-BSA-D3 on Aβ1–42 and NIR irradiation on the aggregation process. Aβ1–42 was incubed in the presence or absence of sAuNPs-BSA-D3 and irradiated for 1 h. The Fluorescence intensity of Aβ1–42 measured after 72 h of incubation, showing the that after irradiation in the presence of sAuNPs-BSA-D3 there is a marked reduction in fluorescence signal. *** *p* < 0.001 (*n* = 2) in triplicate. The fluorescence intensity is normalized with respect to the non irradiated sample.
