# Supplementary material for: A Degradable Nanosystem Based on Small Gold Nanoparticles and Albumin for Amyloid Aggregation Inhibition
Source: Pharmaceutics. 2026 Apr 19;18(4):504. doi: 10.3390/pharmaceutics18040504 (PMC13119133; doi:10.3390/pharmaceutics18040504)
Supplement: Supplementary file 1 [file pharmaceutics-18-00504-s001.zip › pharmaceutics-4264557-supplementary.pdf]

Supporting Information

Article

A Degradable Nanosystem Based on Small Gold Nanoparticles and Albumin for Amyloid Aggregation Inhibition

Matias Levio [1,†], Francisco Rossel Carrera [1,2,†], Fredys Sánchez Hoyos [1,3], Maycol Huerta [2], Carlos Alamos [1,3], Rodrigo Vásquez-Contreras [2], Marcelo J. Kogan [1,3,*] and Eyleen Araya Fuentes [2,*]

[1] Facultad de Ciencias Quimicas y Farmacéuticas, Universidad de Chile, Sergio Livingstone 1007, Santiago 8380492, Chile; matias.levio@ug.uchile.cl (M.L.); frosselcarrera@gmail.com (F.R.C.); fsanchezh@unicartagena.edu.co (F.S.H.); labkogan@gmail.com (C.A.)

[2] Departamento de Ciencias Quimicas, Facultad de Ciencias Exactas, Universidad Andres Bello, Republica 275, Santiago 8370146, Chile; m.huertamatus@uandresbello.edu (M.H.); rvasquez1982@gmail.com (R.V.-C.)

[3] Advanced Center for Chronic Diseases (ACCDiS), Facultad de Ciencias Quimicas y Farmaceuticas, Universidad de Chile, Dr. Carlos Lorca Tobar 964, Santiago 8380494, Chile

* Correspondence: mkogan@ciq.uchile.cl (M.J.K.); eyleen.araya@unab.cl (E.A.F.)

† These authors contributed equally to this work.

1-Characterization of the peptide D3

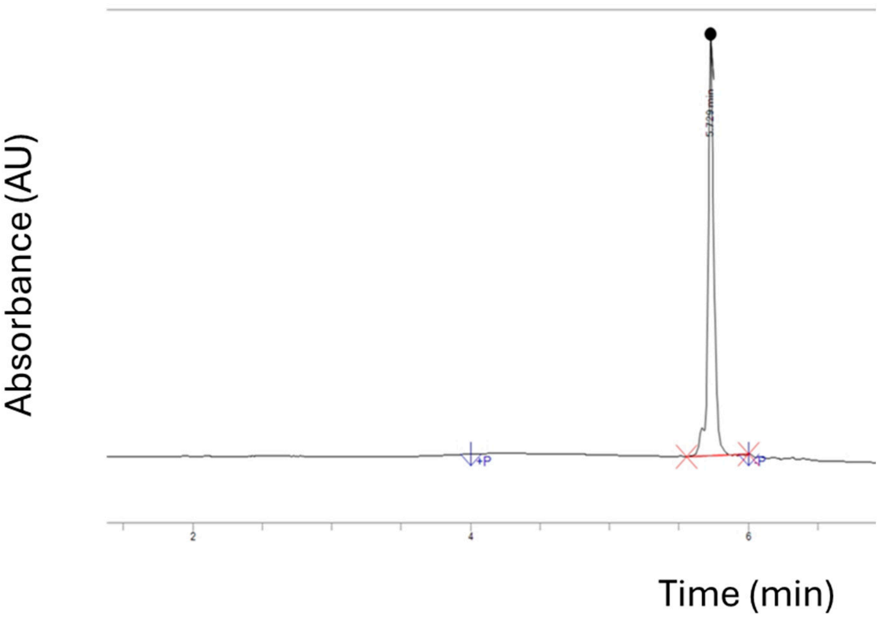

Figure S1A. HPLC analysis of the obtained peptide D3. Gradient water–acetonitrile (0-100%, 15 min), flux 1 ml/min. Retention time 5.7 min. Purity obtained: 97%.

UPLC-Ms mass spectrum of D3 ionic fragments

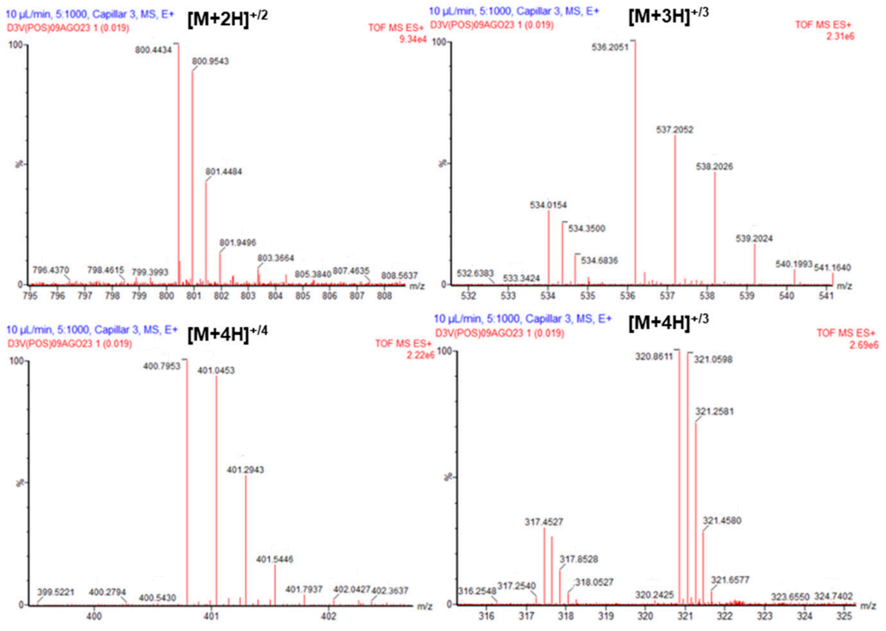

Figure S1B. Mass spectrum of the peptide D3: ionic fragments.

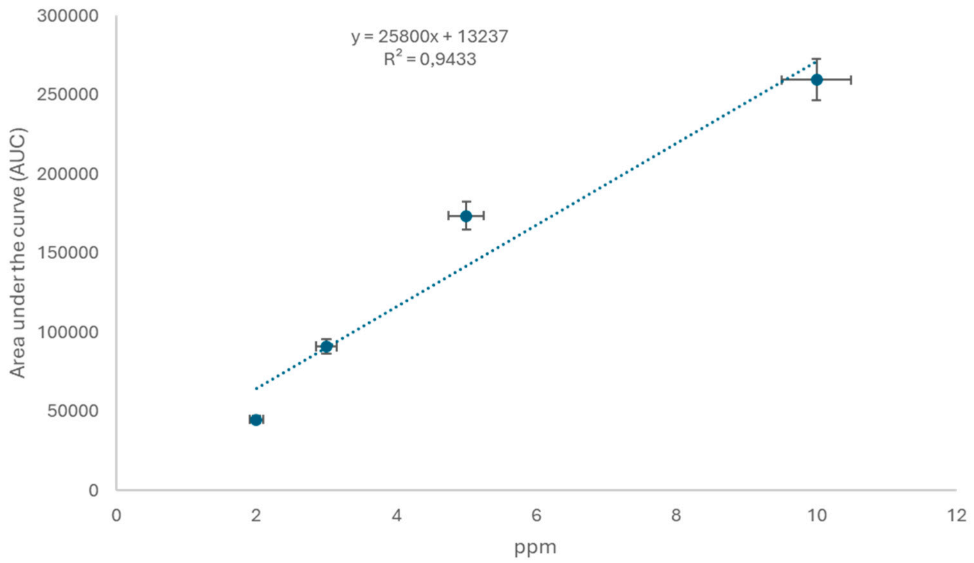

Figure S2. The calibration curve for peptide D3 obtained from the area under the chromatographic peak corresponding to the peptide signal. UPLC analysis was performed using a reverse-phase C18 column with a 0–100% acetonitrile gradient (water–ACN system) over 10 min. The retention time of peptide D3 was approximately 6 min.

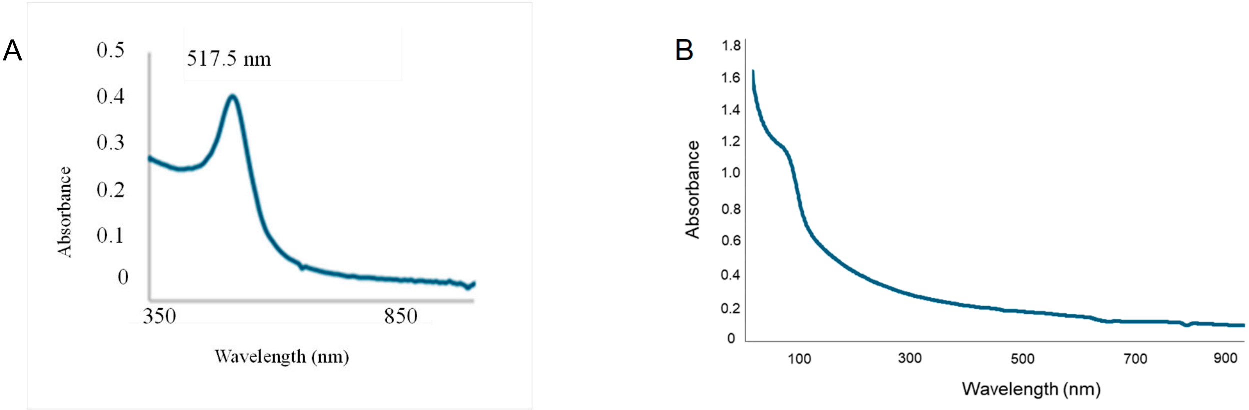

Fig S3. A) UV-Vis-NIR spectrum of sAuNPs. B) UV-Vis-NIR spectrum of BSANPs.

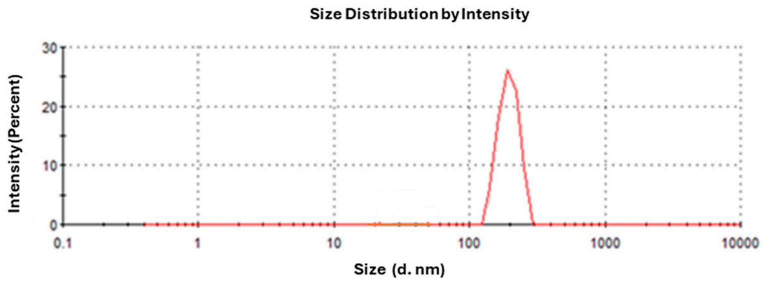

Figure S4. Size distribution of BSANPs (DLS) obtained via desolvation method.

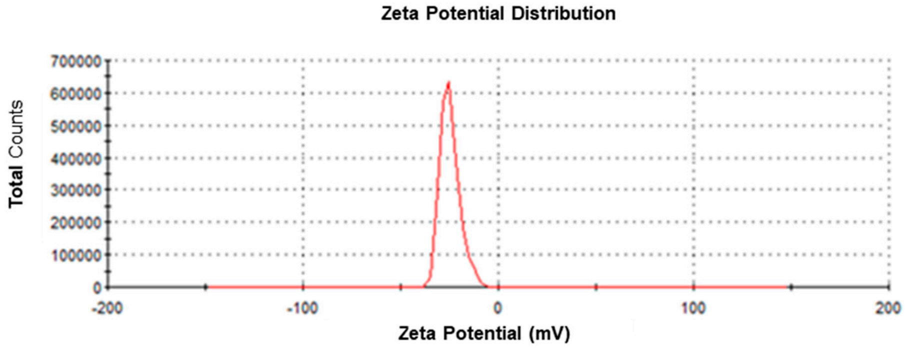

Figure S5. Zeta potential of BSANPs (DLS) obtained via desolvation method.

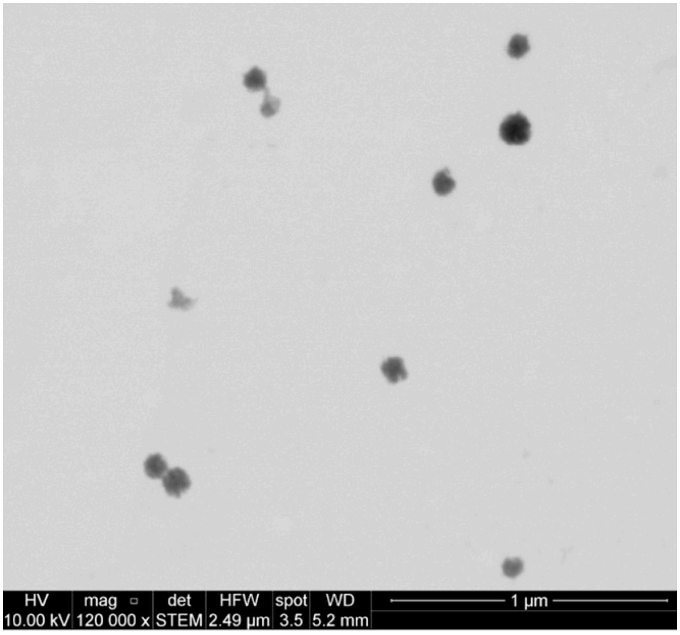

Figure S6. STEM image of BSANPs obtained via desolvation method.

Table S1. Determination of the efficiency of encapsulation of gold nanoparticles in the nanosystem f-sAuNPs-BSANPs.

The samples of f-sAuNPs-BSANPs were quantified via atomic absorption using Analytik Jena ContrAA 800. A calibration curve from commercially available 1000 mg/L of Au single-element standard (Merck, Darmstadt, Germany), appropriately diluted with 0.5% HCl (analytical grade, Merck), was prepared, ranging from 300 pg to 3750 pg of gold. The operation conditions are described in the next table. The efficiency of encapsulation was obtained by the difference between the initial concentration of f-sAuNPs and the concentration incorporated in the pellets.

Operation conditions for Au determination by GFAAS

Signal processing: Peak area; Read time: 5 s<br>Background correction: Zeeman effect<br><br>Wavelength—242.80 nm<br>Slit—0.7 nm				
Step	Temp (°C)	Ramp (°C/s)	Hold (s)	Time (s)
Drying	80	6	20	27.5
Drying	90	3	20	23.3
Drying	110	5	10	14.0
Pyrolysis	350	50	20	24.8
Pyrolysis	500	300	10	10.5
Gas adaption	500	0	5	5.0
Atomize	2000	1500	3	4.0
Cleaning	2450	500	4	4.9

Table S2. Initial temperature, final temperature and temperature increase of BSANPs and f-sAuNPs-BSA-D3 after 1 h of 808 nm NIR laser irradiation.

Sample	Initial temperature	Final temperature	Temperature difference
BSANPs	14°C	14°C	0°C
**f-sAuNPs-BSA-D3**	14°C	19°C	5°C

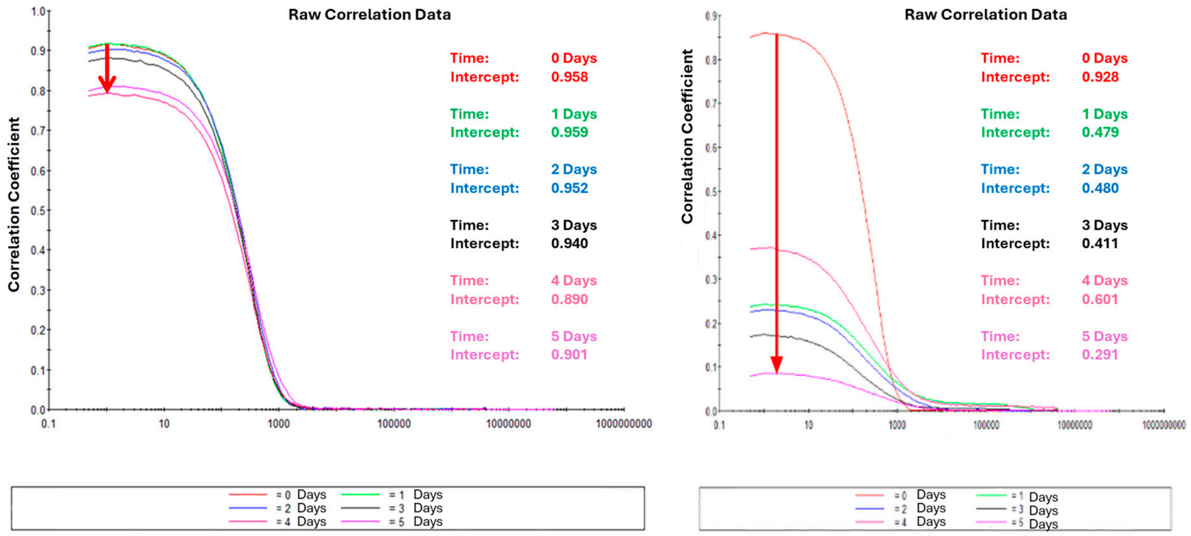

Figure S7. Effect of trypsin on f-sAuNPs-BSANPs-D3 nanosystem on correlograms obtained by DLS. Nanosystems incubated with 0.00001 (a) and 0.001% (b) of trypsin concentrations to simulate systemic proteolytic conditions and high-activity compartments, respectively. Samples were incubated at 37 °C in buffer PBS at pH=7.4.

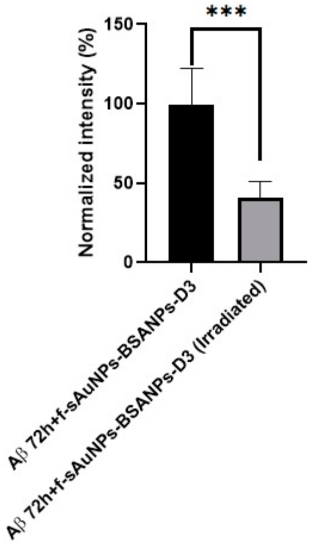

Figure S8. Effect of sAuNPs-BSA-D3 on $A\beta_{1-42}$ and NIR irradiation on aggregation process. $A\beta_{1-42}$ was incubated in presence or absence of sAuNPs-BSA-D3 and irradiated for 1h. Fluorescence intensity of $A\beta_{1-42}$ measured after 72 h of incubation, showing that after irradiation in presence of sAuNPs-BSA-D3 there is marked reduction in fluorescence signal. *** p<0.001 (*n*=2) in triplicate. Fluorescence intensity is normalized with respect to non-irradiated sample.
